# Age‐related changes to macrophage subpopulations and TREM2 dysregulation characterize attenuated fracture healing in old mice

**DOI:** 10.1111/acel.14212

**Published:** 2024-06-02

**Authors:** Daniel Clark, Sloane Brazina, Ted Miclau, Sangmin Park, Christine L. Hsieh, Mary Nakamura, Ralph Marcucio

**Affiliations:** ^1^ Center for Craniofacial Regeneration University of Pittsburgh School of Dental Medicine Pittsburgh Pennsylvania USA; ^2^ Department of Periodontics and Preventive Dentistry University of Pittsburgh School of Dental Medicine Pittsburgh Pennsylvania USA; ^3^ Department of Obstetrics and Gynecology University of California Davis Sacramento California USA; ^4^ Orthopaedic Trauma Institute, Department of Orthopaedic Surgery, School of Medicine University of California San Francisco, Zuckerberg San Francisco General Hospital San Francisco California USA; ^5^ Division of Rheumatology, Department of Medicine University of California San Francisco, San Francisco VA Health Care System San Francisco California USA

**Keywords:** fracture healing, macrophage, sequencing analysis RNA

## Abstract

Fracture healing complications increase with age, with higher rates of delayed unions and nonunions and an associated increase in morbidity and mortality in older adults. Macrophages have a dynamic role in fracture healing, and we have previously demonstrated that age‐related changes in macrophages are associated with attenuated fracture repair in old mice. Here, we provide a single cell characterization of the immune cells involved in the early phase of fracture healing. We show that there were multiple transcriptionally distinct macrophage subpopulations present simultaneously within the healing tissue. Fracture healing was attenuated in old mice compared to young, and macrophages from the fracture callus of old mice demonstrated a pro‐inflammatory phenotype compared to young. Interestingly, Trem2 expression was decreased in old macrophages compared to young. Young mice lacking Trem2 demonstrated attenuated fracture healing and inflammatory dysregulation similar to old mice. Trem2 dysregulation has previously been implicated in other age‐related diseases, but its role in fracture healing is unknown. This work provides a robust characterization of the macrophage subpopulations involved in fracture healing, and further reveals the important role of Trem2 in fracture healing and may be a potential driver of age‐related inflammatory dysregulation. Future work may further examine macrophages and Trem2 as potential therapeutic targets for management of fracture repair in older adults.

## Graphical abstract

Single‐cell RNAseq analysis demonstrated multiple transcriptionally distinct macrophage subpopulations within the fracture callus. Further analysis revealed significant age‐related changes across these macrophage subpopulations. We identified TREM2 as an important immunoregulator on macrophages during fracture healing and demonstrated age‐related dysregulation of TREM2 may be an important contributor to age‐related attenuated fracture repair.

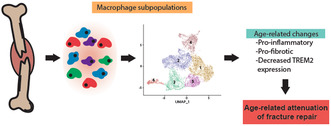

**Abbreviations**
IFN‐γ: interferon‐gamma
IL‐1α: interleukin 1‐alpha
iNOS: inducible nitric oxide synthase
TNFα: tumor Necrosis factor‐alpha
TREM2: triggering receptor expressed on myeloid cells 2

## 1 INTRODUCTION

A dysregulated inflammatory response has been characterized as a central pathological change that occurs with increased age. This pathological change has been described as “inflammaging”, which characterizes a low‐grade chronic inflammatory environment that increases with age and contributes to the myriad of pathologies and conditions associated with aging (Xia et al., 2016). While causative mechanisms have not been fully elucidated, dysregulation of the innate immune response appears to contribute significantly to the inflammaging phenotype (Franceschi et al., 2018).

One condition that is negatively impacted by increasing age is fracture healing. Older adults demonstrated higher rates of nonunions and delayed unions compared to young adults (Foulke et al., 2016) (Office of the Surgeon General (US), 2004). Such healing complications result in increased rates of morbidity and mortality following fractures in older adults (Civinini et al., 2019) (Guzon‐Illescas et al., 2019). Given that the immune response is critical for successful fracture repair (Baht et al., 2018), a better understanding of how age‐related inflammatory dysregulation impacts fracture healing in older adults may lead to better treatment outcomes for the aging adult population.

In investigating age‐related inflammatory dysregulation, our group, and others, have previously demonstrated attenuated fracture healing outcomes in old mice compared to young and have further implicated the contribution of the aging immune response in the perturbed healing. (Lu et al., 2005) (Lopas et al., 2014) (Xing, Lu, Hu, Miclau, et al., 2010). We have shown that fracture healing was significantly improved after transplanting bone marrow from young mice into old mice prior to fracture (Xing, Lu, Hu, Miclau, et al., 2010). In this model, the osteoblasts and chondrocytes were derived from the host and the immune cells were derived from the young donor, which reduced inflammation and stimulated bone fracture healing compared to age‐matched control chimeras. This was subsequently confirmed using a murine parabiosis model of young and old mice (Baht et al., 2015). In these studies, the contribution of specific immune cell types to the aging phenotype was not studied.

Towards an improved cellular understanding of the age‐related perturbation of the immune response during fracture healing, we have focused our work on the macrophage. Macrophages are essential for successful fracture healing and are a heterogenous population with diverse phenotypes and functions that change throughout the healing process (Alexander et al., 2011; Batoon et al., 2017; Xing, Lu, Hu, Yu, et al., 2010). We have previously shown that macrophages present in the fracture callus of old mice have a pro‐inflammatory phenotype and are transcriptionally distinct from macrophages in the callus of young mice. By preventing migration of macrophages into the fracture callus of old mice, fracture healing was significantly improved (Clark et al., 2020). Interestingly, there was no beneficial effect of inhibiting macrophage influx into the callus in young mice. Given the phenotypic complexity and diverse roles of macrophages in both propagating and resolving inflammation, it is not clear what subpopulation of macrophages are critical for fracture healing or what was driving the age‐related changes.

In this study, we used single cell RNA sequencing (scRNAseq) to analyze the immune cell populations involved in fracture healing. We further analyzed the macrophage populations in old and young mice and identified age‐related changes in multiple subpopulations of macrophages that may contribute to the inflammatory dysregulation and perturbed fracture healing outcomes in older animals. We determined that a key macrophage receptor, triggering receptor expressed on myeloid cells 2 (TREM2), has an important role in fracture healing, and age‐related dysregulation of TREM2 may contribute to the altered activity of macrophages in aged mice, the inflammatory dysregulation and the subsequent delayed fracture healing observed in these animals.

## 2 RESULTS

### 2.1 Attenuated fracture repair in old mice is associated with local inflammatory dysregulation

**FIGURE 1:**
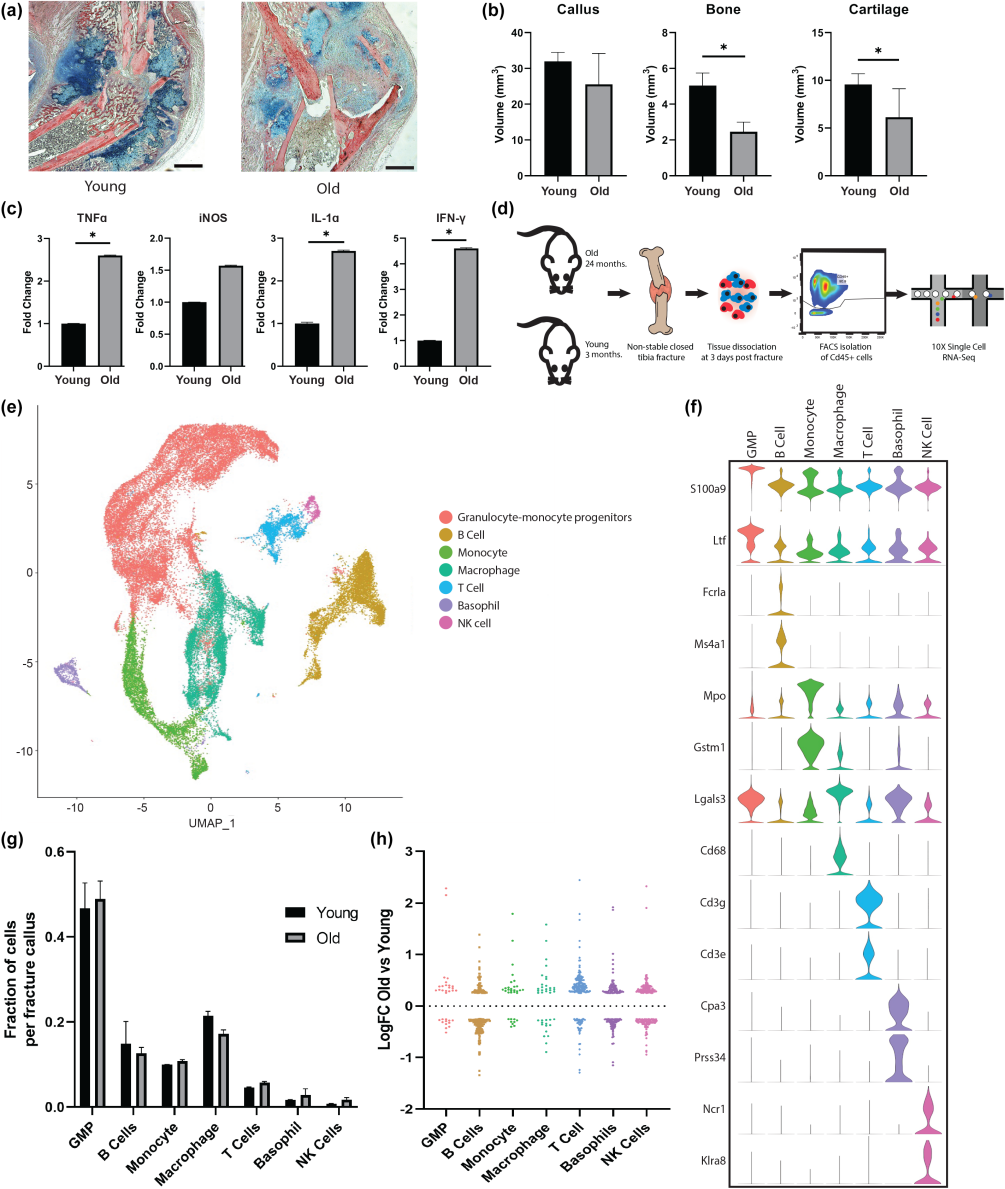
Attenuated fracture repair in old mice is associated with local inflammatory dysregulation, and single cell analysis reveals a heterogenous immune cell population infiltrating the early fracture callus. (a) Representative histological sections of fracture callus (10 days post fracture) in old (24 month) and young (4 month) mice using Hall Brunt Quadruple Stain (HBQ) (bone = red, cartilage = blue) (scale bar = 1 mm). (b) Stereological analysis of serial histological sections quantifies volume of the fracture callus and volume of bone and cartilage with the callus at 10 days post fracture in old and young mice (*n* = 6/group). (c) qPCR analysis of inflammatory cytokine expression within the fracture callus at 5 days post fracture in old and young mice (*n* = 5/group). (d) Schematic of the single cell RNA sequencing experiment that analyzed Cd45+ immune cells from the fracture callus of old and young mice. (e) Uniform Manifold Approximation and Projection (UMAP) analysis of the transcriptome of 42,070 cells from the fracture callus of old and young mice. Immune cell types were determined based on analysis of their characteristic gene expression profiles. (f) Violin plots demonstrating the expression of characteristic marker genes used to define each cell type (monocytes, macrophages, granulocyte‐monocyte progenitors (GMP), T cells, B cells, basophils, NK cells). (g) Bar plot demonstrating the fraction of cells within each cell type for old compared to young mice (mean ± SD). (h) Plot showing the log fold change of all differentially expressed genes in each cell types as a function of age (logFC>0.25 and gene expressed in 10% or more of cells within cluster). *(*p* < 0.05).

To begin, we analyzed fracture healing in old and young mice. Closed, non‐stable fractures of the tibia were created in old (24 months) and young (4 months) C57BL/6 mice (*n* = 6/group). Fracture healing was evaluated via stereology at 10 days post fracture when new bone tissue is expected to be present (Clark et al., 2020) (Marmor et al., 2020). Serial histological sections of the fracture callus were prepared (Figure 1a), and the volume of the bone and cartilage within the callus was analyzed. Old mice had significantly less bone and cartilage volume within the fracture callus compared to young mice (*p* < 0.05) (Figure 1b). Next, relative expression of pro‐inflammatory cytokines in the fracture callus of old and young mice were determined using quantitative real‐time PCR (qPCR). Tibia fractures were created, and the callus was isolated at 5 days post fracture. Old mice had significantly higher expression of TNFα, IL‐1α, and IFN‐γ within the callus compared to young mice (*p* < 0.05) (Figure 1c). Here, the local inflammatory environment in old mice was associated with attenuated fracture repair, which is characteristic of the pathological age‐related inflammatory dysregulation that has been well described in humans and animal models (Kennedy et al., 2014).

### 2.2 Heterogenous immune cell populations infiltrate the early fracture callus

The importance of immune cells in the fracture healing process is well appreciated (Baht et al., 2018), so we wanted to assess how aging affect immune cells during fracture healing. To accomplish this, we used scRNAseq to characterize the immune cell populations infiltrating the fracture callus in young and old mice (Figure 1d). At 3 days post fracture, which is the peak of immune cell infiltration into the fracture callus (Clark et al., 2020), Cd45+ cells were isolated from the fracture callus of old (*n* = 2) and young (*n* = 2) mice via flow cytometry. The sorted immune cells were prepared in a single cell suspension and library preparation was performed using the Chromium Single Cell 3′ Reagent Version 3 Kit (10X Genomics). Libraries were then sequenced on an Illumina HiSeq2500. Cells were filtered, normalized, and clustered. Cells were removed from analysis according to thresholds described in the methods sections. The transcriptome of a total of 42,070 cells were included in the final analysis. Transcriptionally similar cells were aggregated into clusters and seven Cd45+ immune cell types were identified within the fracture callus based on characteristic gene expression profiles (monocytes, macrophages, granulocyte‐monocyte progenitors, T cells, B cells, basophils, and NK cells) (Figure 1e,f). The number of cells within each cell type was not significantly different in old compared to young mice (Figure 1g), but significantly transcriptional differences were observed. Differentially expressed genes (DEGs) in old mice compared to young were present across each cell type (logFC>0.25 and gene expressed in 10% or more of cells within cluster) (Figure 1h). See Table S1 for list of all age‐related DEGs by cell type.

### 2.3 Macrophage subpopulations within the fracture callus demonstrate transcriptional heterogeneity

**FIGURE 2:**
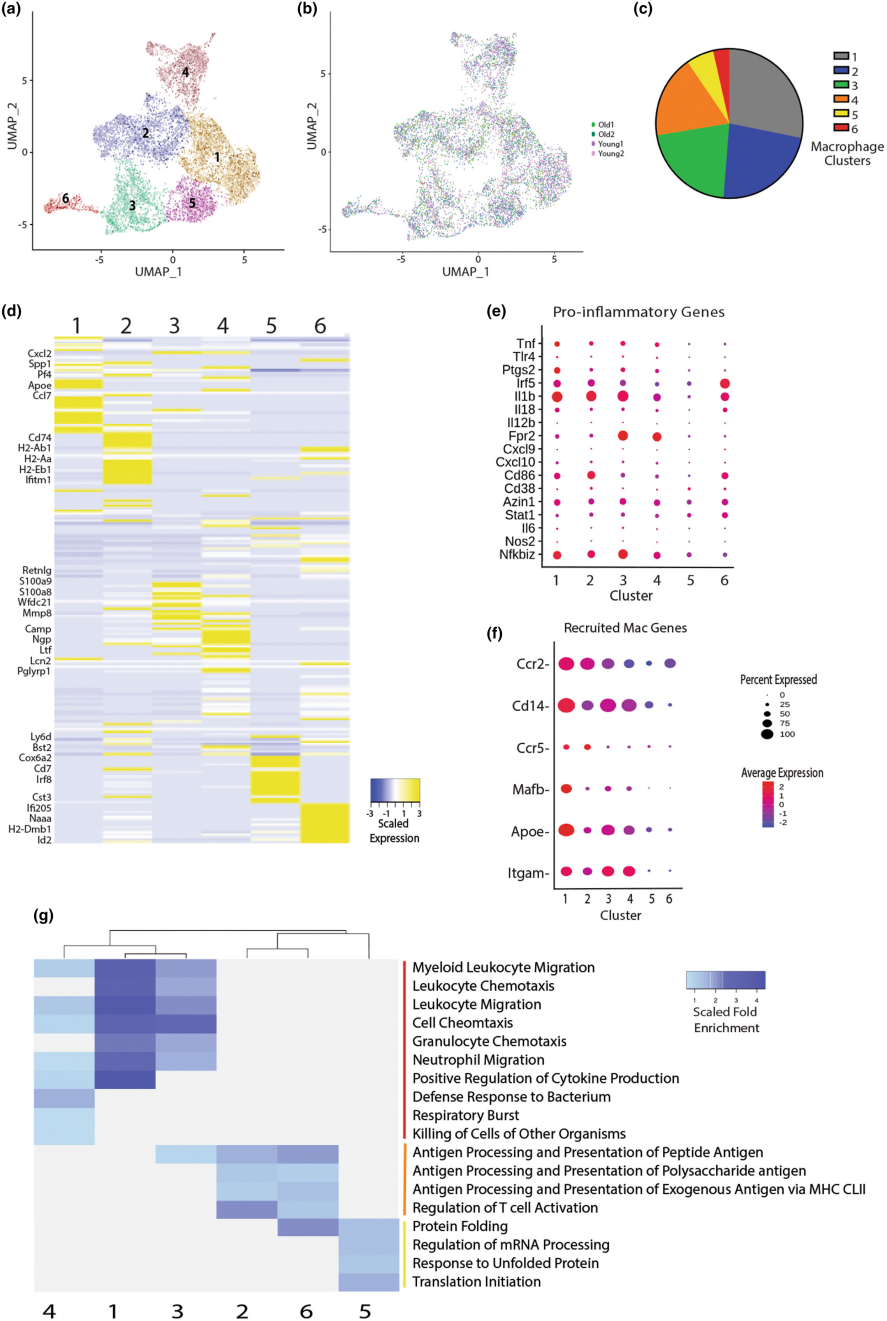
Macrophage subpopulations within the fracture callus demonstrate transcriptional heterogeneity. The macrophage cell cluster identified in the initial analysis of CD45+ cells from the fracture callus of old and young mice was re‐analyzed and re‐clustered, resulting in the identification of six transcriptionally distinct macrophage subpopulations (clusters). (a) UMAP demonstrating the six macrophage‐only clusters. (b) UMAP annotated by sample and age group. (c) Pie chart demonstrating the proportion of macrophages within each cluster compared to total macrophages. (d) Heatmap of DEGs across the macrophage clusters. The top 5 genes (by fold change) are listed for each cluster (FDR<0.05, LogFC>0.25). (e, f) Dot plots show the differential expression of pro‐inflammatory genes and recruited macrophage marker genes across all clusters. (g) Gene ontology analysis of the enriched biological processes and pathways across all clusters. Significantly enriched terms (FDR <0.1) are shown. Terms are grouped into recruited macrophages functions (red bar), immune surveillance terms (orange bar), and other terms (yellow bar). Clusters are hierarchically sorted based on the scaled fold enrichment of each GO term.

Macrophages are critical throughout the fracture healing process (Schlundt et al., 2018), and we have previously shown that age‐related changes to macrophages are detrimental to fracture healing (Clark et al., 2020). However, given the functional and phenotypic heterogeneity of macrophages, we wanted to further analyze the macrophage subpopulations in the fracture callus of young and old mice via scRNAseq. A broad characterization of macrophage cell populations was initially identified by the expression of representative cell‐type specific markers (CD68, Lgals3) (Figure 1f). Re‐clustering of this macrophage population identified 6 transcriptionally distinct macrophage subpopulations across old and young mice (Figure 2a–c). The top 5 expressed genes that characterized each macrophage cluster are listed in Figure 2d. See Table S2 for all cluster marker genes for each macrophage subpopulation (FDR<0.05, LogFC>0.25). To understand the relative phenotypic heterogeneity of the macrophages subpopulations, we analyzed the differential expression of pro‐inflammatory genes and recruited macrophage marker genes across the macrophage subclusters (Figure 2e,f). High expression of pro‐inflammatory genes are noted in clusters 1–4, which were clusters that also demonstrated high expression of recruited macrophage marker genes. Increased infiltration of circulating monocyte‐derived macrophages with a pro‐inflammatory phenotype is characteristic of the inflammatory events during the early time points after injury to bone (Baht et al., 2018). Macrophage clusters 5 and 6 appeared transcriptionally distinct from the other clusters with lower expression of pro‐inflammatory genes and recruited macrophage genes (Figure 2e,f). Expression of anti‐inflammatory cytokines genes were largely absent from all clusters (Table S2) and likely reflected the pro‐inflammatory status of the early time point analyzed at 3 days post fracture.

To better understand the general function of each macrophage subpopulation, the biological processes and pathways enriched in each cluster were analyzed. Gene ontology (GO) analysis was performed, and clusters were sorted hierarchically based on the differential enrichment of the biological processes and pathways within each cluster (Figure 2g). Clusters 1, 3, and 4 were enriched for biological processes and pathways associated with recruited macrophage function including immune cell migration and chemotaxis, cytokine production, and respiratory burst, which is consistent with the increased expression of recruited macrophage marker genes in these clusters (Figure 2f). Interestingly, cluster 2 similarly expressed high levels of recruited macrophage marker genes but differentiated from the other recruited clusters with enrichment of biological processes and pathways associated with immune surveillance and antigen presentation. It appears the recruited macrophage populations demonstrate diverse immune and inflammatory functions concurrently during the fracture healing process. The enriched biological processes and pathways for clusters 5 and 6 were distinct from recruited macrophage functions. Cluster 6 was enriched for immune surveillance terms and cluster 5 enriched for terms distinct from all other clusters (Figure 2g). Together, the analysis of the macrophage subpopulations demonstrates that the early fracture callus is characterized by heterogenous macrophage populations with distinct transcriptional profiles.

### 2.4 Identifying age‐related transcriptional changes across macrophage subpopulations

**FIGURE 3:**
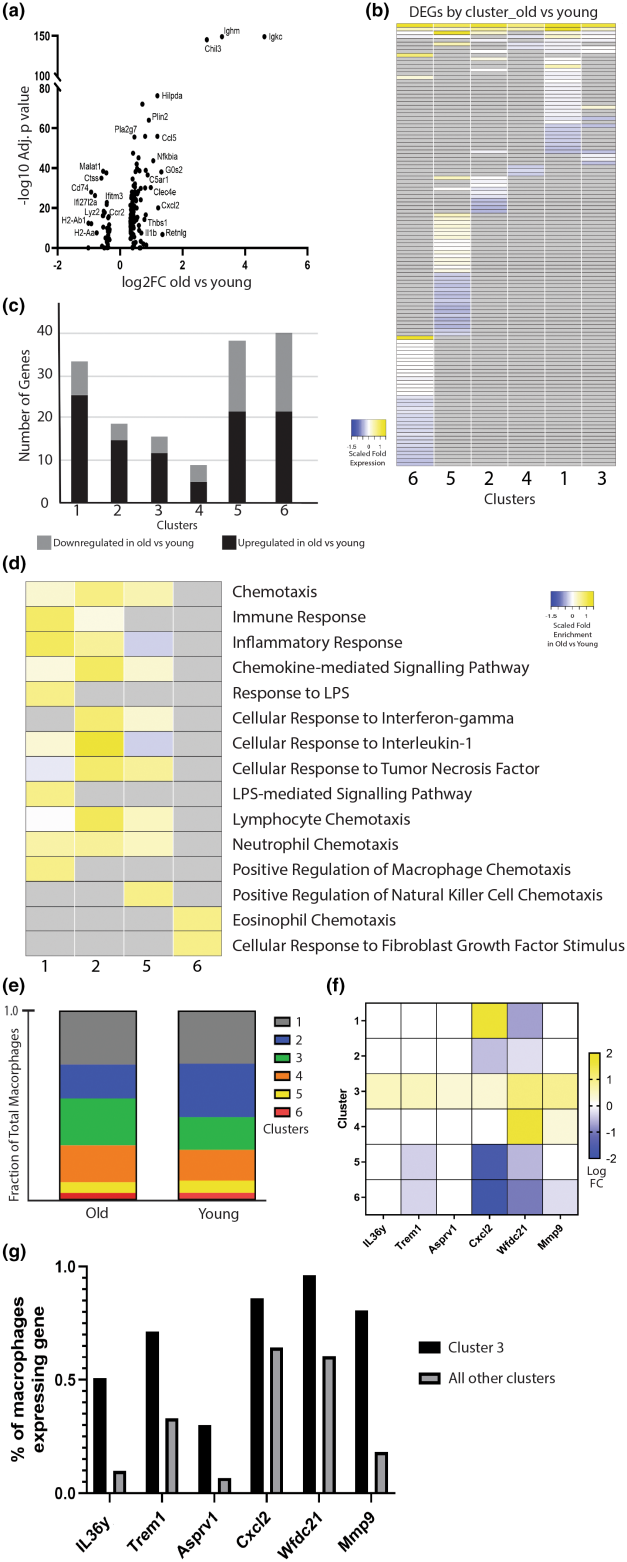
Identifying age‐related transcriptional changes across macrophage subpopulations. Differentially expressed genes in old mice versus young were compared within each macrophage cluster. (a) Volcano plot of DEGs across all macrophage clusters in old mice compared to young mice (logFC>0.1, *p* < 0.05). (b) Heatmap demonstrating the fold change of each age‐related DEG within each cluster. Each row is a different gene that was differentially expressed in at least one cluster. Gray indicates the gene was not significantly changed as a function of age within the given cluster. (c) Bar graph representing the number of DEGs that were up and downregulated in macrophages from old mice compared to young within each cluster. (d) Heatmap of enriched biological processes and pathways associated with the upregulated DEGs within each cluster via GO analysis. Significantly enriched terms (FDR<0.1) are shown. Gray bars indicate the GO term was not enriched with the cluster. (e) Bar graph demonstrating the fraction of macrophages within each cluster compared to total macrophages in the old and young samples. (f) Heatmap demonstrating the logFC of fibrosis‐associated macrophage marker genes across all macrophage clusters. (g) Bar graph demonstrating the proportion of cells expressing the fibrosis‐associated macrophage marker genes within cluster 3 compared to all other clusters.

We next wanted to understand if age had a differential effect across the macrophage subpopulations. Differential gene expression analysis was performed to identify the age‐related transcriptional changes in the macrophage subpopulations. Analysis of DEGs across all macrophage clusters demonstrated 165 genes that were significantly differentially expressed in macrophages from old mice compared to young (130 upregulated, 35 downregulated) (log2FC>0.1, *p* < 0.05) (Figure 3a; Table S3). Genes significantly upregulated in old macrophages included inflammatory response related genes (Il1β, Chil3, Nfkbiz, Thbs1), immune cell chemotaxis genes (Ccl2, Ccl5, Cxcl2, CxCl3), apoptosis regulator genes (Cflar, Mcl1, Rack1), and multiple ribosomal protein genes that have been shown to be dysregulated with age (Rpl8, Rpl10, Rps2, Rps20) (Frenk & Houseley, 2018) (Ximerakis et al., 2019). Further analysis revealed the age‐related DEGs within each macrophage subpopulation (Figure 3b,c; Table S3). Interestingly, the subpopulations of macrophages are affected by age in transcriptionally unique ways, with each subpopulation of macrophages demonstrating a largely unique set of DEGs (Figure 3b; Table S3). Macrophages in clusters 1 and 2 appeared to have the most pro‐inflammatory transcriptional changes as a function of age with an upregulation of the immunoregulatory and pro‐inflammatory genes (IL‐1β, NfKbia, Ccl2, Ccl4, Ccl5, Cxcl2) from old mice compared to young within the same clusters (Table S3). Macrophages in clusters 1, 5, and 6 had the most DEGs as a function of age, while age appeared to have less of an effect on the transcriptome of macrophages in clusters 3 and 4 (Figure 3c).

GO analysis was performed on the genes upregulated as a function of age within the macrophage subpopulations. Genes that were upregulated in old macrophages within clusters 1 and 2 were enriched for pro‐inflammatory processes such as neutrophil and lymphocyte chemotaxis and inflammatory and immune response (Figure 3d). The upregulated DEGs in clusters 5 and 6 were not enriched to the same extent in the pro‐inflammatory pathways and cellular processes. There was no enriched pathway or biological process within cluster 3 and 4 due to the low number of DEGs within the clusters.

The fraction of macrophage subpopulation remained relatively consistent in old and young mice, with the most noticeable change being an expansion of cluster 3 and a reduction of cluster 2 in old macrophages compared to young (Figure 3e). Macrophages in cluster 3 had an enrichment for genes that were shown to be markers of a fibrosis‐associated macrophage subpopulation (Il36γ, Trem1, Asprv1, Cxcl2, Wfdc21, Mmp9) (Figure 3f,g). This gene set was previously shown to characterize macrophage subset involved in fibrosis during wound healing (Sommerfeld et al., 2019). Such an expansion of a fibrosis‐associated macrophage subpopulation as seen here may be detrimental during fracture healing where a more skeletogenic response is needed for successful healing outcomes.

### 2.5 Validation of age‐related transcriptional changes in macrophages

**FIGURE 4:**
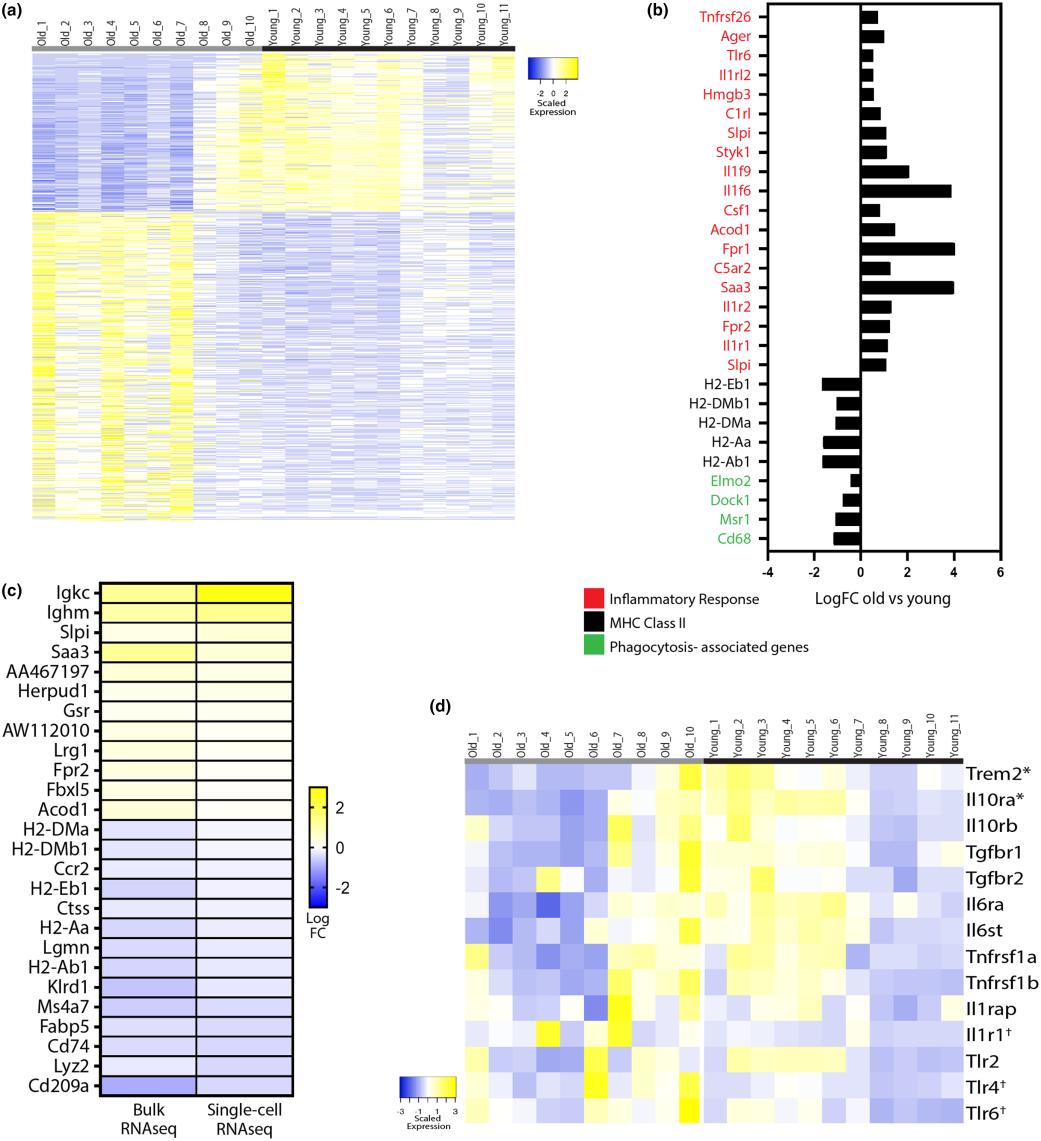
Validation of age‐related transcriptional changes in macrophages via bulk RNAseq. (a) Heatmap of DEGs in macrophages derived from old mice (*n* = 10) compared to young (*n* = 11) via bulk RNAseq (FDR<0.1, LogFC>0.25). (b) Bar graph of the LogFC of selected DEGs in macrophages from old mice compared to young (FDR<0.1). (c) Heatmap of the LogFC of DEGS in macrophages from old mice compared to young that were similarly significantly differentially expressed in the bulk RNAseq and scRNAseq data sets. (d) Heatmap of selected genes encoding receptors involved in inflammatory pathways across each sample in the bulk RNAseq data set. *significantly downregulated in old macrophages compared to young, Ɨsignificantly upregulated in old macrophages compared to young.

We next sought to further validate the age‐related transcriptional changes in the macrophages from the fracture callus of old mice using an expanded sample size. We incorporated and reanalyzed a previously generated data set from our group (Clark et al., 2020). Bulk RNA sequencing analysis was performed on macrophages isolated from the fracture callus of old (*n* = 10) and young (*n* = 11) mice. The same fracture model was utilized, and macrophages were isolated from the fracture callus via flow cytometry at the same time point (Day 3 post fracture). Bulk RNA sequencing validated the robust transcriptional differences between macrophages from old and young mice (Figure 4a; Table S4). The heatmap shows the DEGs in old macrophages compared to young (FDR<0.1, LogFC>0.25), and illustrates the discrimination of the age groups based on differential gene expression (Figure 4a). There were 1132 genes differentially expressed in the old macrophages compared to young, with the majority of genes (749) demonstrating upregulated expression with increased age. The list of DEGs contained genes that have previously demonstrated age‐related changes across cell and tissue types (Grolleau‐Julius et al., 2008; Herrero et al., 2001; Ximerakis et al., 2019). Such age‐related transcriptional changes identified here include upregulation of genes associated with the inflammatory response, consistent with the inflammatory dysregulation that is associated with increased age (Figure 4b). Additionally, decreased expression of MHC class II genes and phagocytosis‐associated genes was observed (Figure 4b). There was also an age‐related upregulation of fibrosis‐associated genes Il36γ and Asprv1 (Table S4) that we had similarly identified in a macrophage subpopulation (cluster 3) in the single cell analysis (Figure 3f,g).

We next compared the DEGs in macrophages from old mice derived from both the single cell and bulk RNAseq data sets. From this, a list of 26 genes were identified that were similarly significantly differentially expressed in both data sets (Figure 4c; Table S4). This list provides a robust characterization of the aging gene signature of macrophage within the context of bone injury. This macrophage aging signature includes genes that were previously shown to have age‐related transcriptional changes, as well as a novel set of genes to characterize age‐related changes to macrophages.

The bulk RNAseq dataset was further used to analyze the differential expression of genes that encode receptors involved in pro‐ and anti‐inflammatory pathways. We hypothesized that age‐related dysregulation of these receptor genes may contribute, in part, to the age‐related inflammatory dysregulation that is characteristic of aging (Xia et al., 2016). We found that anti‐inflammatory pathway receptor genes, Trem2 and IL10ra, were downregulated in macrophages from old mice while pro‐inflammatory pathway receptor genes IL1r1, Tlr4, Tlr6 demonstrated increased expression in old macrophages (*p* < 0.05; Figure 4d). An upregulation of pro‐inflammatory pathway receptors along with a down regulation of anti‐inflammatory pathway receptors may perturb the macrophage from being able to respond to its local environment appropriately, and it may contribute to the pro‐inflammatory phenotype in macrophages from old mice that we have described here thus far.

Validation of the age‐related decrease in Trem2 expression in the macrophages of old mice during fracture repair. We were interested in further validating the age‐related decreased expression of Trem2 in macrophages during fracture repair. Trem2 is expressed on macrophages and upon activation downregulates the inflammatory response, in part, through downregulating pro‐inflammatory cytokine expression (Turnbull et al., 2006). Trem2 association with other age‐related diseases has been previously shown. Variants of Trem2 have been shown to be strongly associated with Alzheimer's Disease (AD) risk (Carmona et al., 2018). In Nasu‐Hakola disease, a rare loss of function mutation to Trem2 results in early on set dementia as well as polycystic osseous lesions with recurrent bone fractures (Dardiotis et al., 2017). While the role of TREM2 in AD and related dementias has been well studied, little attention has been given to the role of TREM2 in the skeletal system.

**FIGURE 5:**
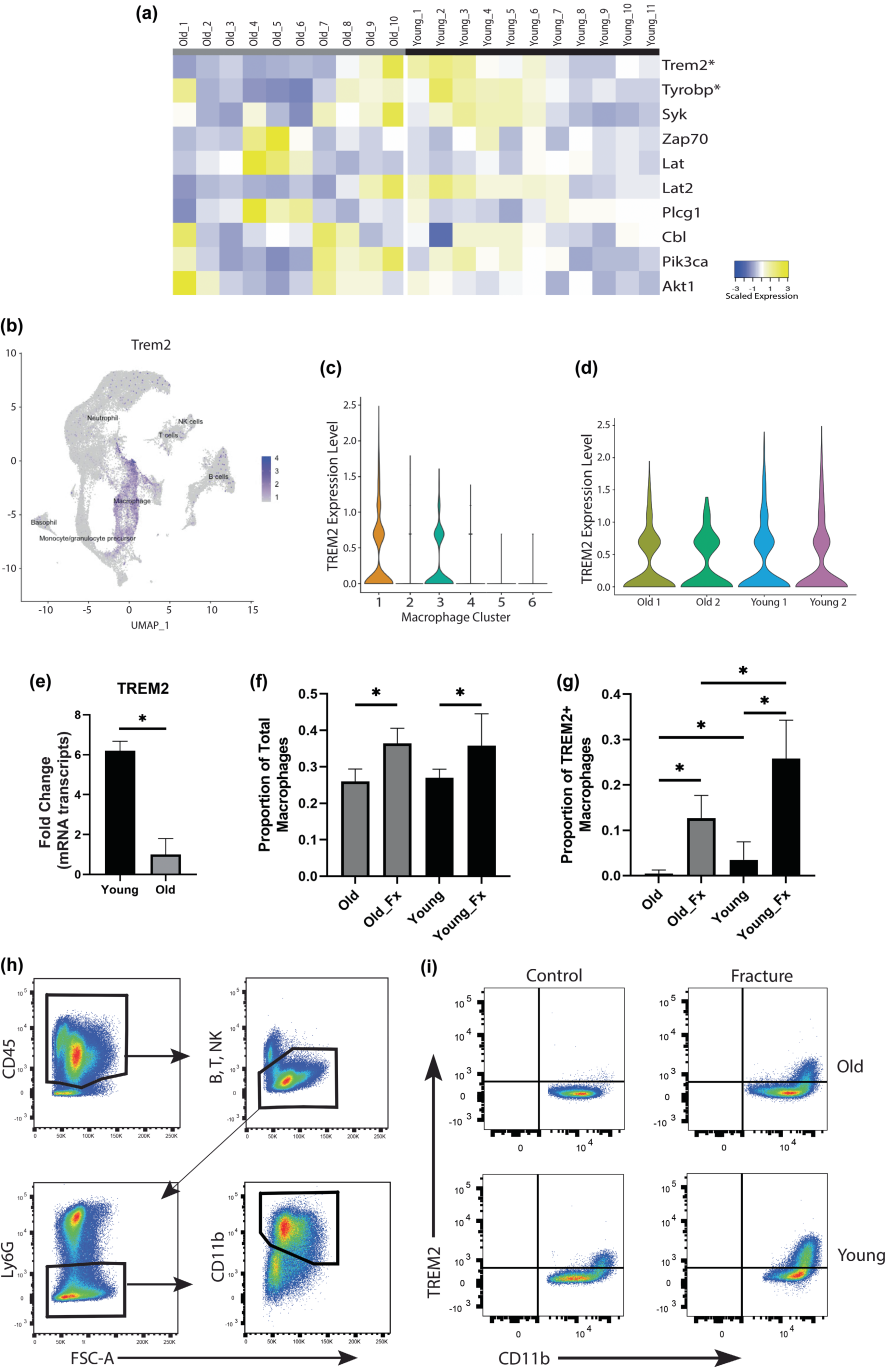
Validation of the age‐related decrease in Trem2 expression in the macrophages of old mice during fracture repair. (a) Heatmap of genes involved in the Trem2 signaling pathway expressed by macrophages isolated from the fracture callus of old and young mice and analyzed via bulk RNAseq. (b) UMAP demonstrating Trem2 expression across the Cd45+ immune cells isolated from the fracture callus of old and young mice and analyzed by single cell RNAseq. (c) Violin plot demonstrating Trem2 expression across the macrophage clusters. (d) Violin plot demonstrating Trem2 expression by sample and age group within all macrophages. (e) qPCR analysis of Trem2 expression within the fracture callus at 5 days post fracture in old and young mice (*n* = 5/group). (f) Bar plot demonstrating the proportion of macrophages (CD11b+) of total immune cells isolated from the fractured tibia (Fx) and contralateral control tibia in old and young mice (*n* = 8/group). (g) Bar plot demonstrating the proportion of TREM2+ macrophages of total macrophages isolated from the fractured tibia (Fx) and contralateral control tibia in old and young mice as analyzed via flow cytometry (*n* = 8/group). (h) Representative gating strategy used for macrophage identification. (i) Representative gating strategy used to identify the proportion of Trem2+, CD11b+ macrophages *(*p* < 0.05).

In further examining the signaling pathway of Trem2, we observed that only Trem2 expression and its co‐receptor, Tyrobp, were downregulated as a function of age in the bulk RNAseq data set (Figure 5a). The expression of other genes involved in the downstream signaling pathway of Trem2 are listed on the heatmap and were unaffected by age, demonstrating that age does not globally effect this signaling pathway but was limited to expression of the receptor and co‐receptor. The scRNAseq analysis demonstrated that Trem2 expression was largely restricted to macrophage populations (Figure 5b). Additionally, Trem2 expression was limited to macrophage clusters 1 and 3 (Figure 5c). Across all macrophage clusters, a trend for increased Trem2 expression was noted in young mouse samples compared to old (Figure 5d).

We next sought to further validate an age‐related decrease in Trem2 activity during fracture healing. Analysis of the callus tissue at 5 days post fracture via qPCR demonstrated a significant decrease in Trem2 gene expression within the healing callus of old mice compared to young (*n* = 5/group) (Figure 5e). Additionally, flow cytometry was used to characterize Trem2 expression on macrophages within the fracture callus of old and young mice (*n* = 8/group). The number of CD11b + macrophages was increased in the fracture callus compared to the contralateral non‐fractured control tibia. The number of macrophages in old versus young mice did not differ in the fracture callus or in the control (Figure 5e). Trem2+, Cd11b + macrophages demonstrated a significant increase in the fracture callus compared to the contralateral non‐fractured control tibia in both old and young mice. The quantity of Trem2+, Cd11b + macrophages was greater in the fracture callus and control tibia of young mice compared to old (Figure 5f,h). The gating strategy used is represented in Figure 5g and shows selection for Cd45+, Cd11b + macrophages while gating out neutrophils (Ly6G+), T cells (Cd3+), B cells (B220+), and NK cells (NK1.1).

### 2.6 Inflammatory dysregulation and delayed fracture healing in Trem2‐null mice

**FIGURE 6:**
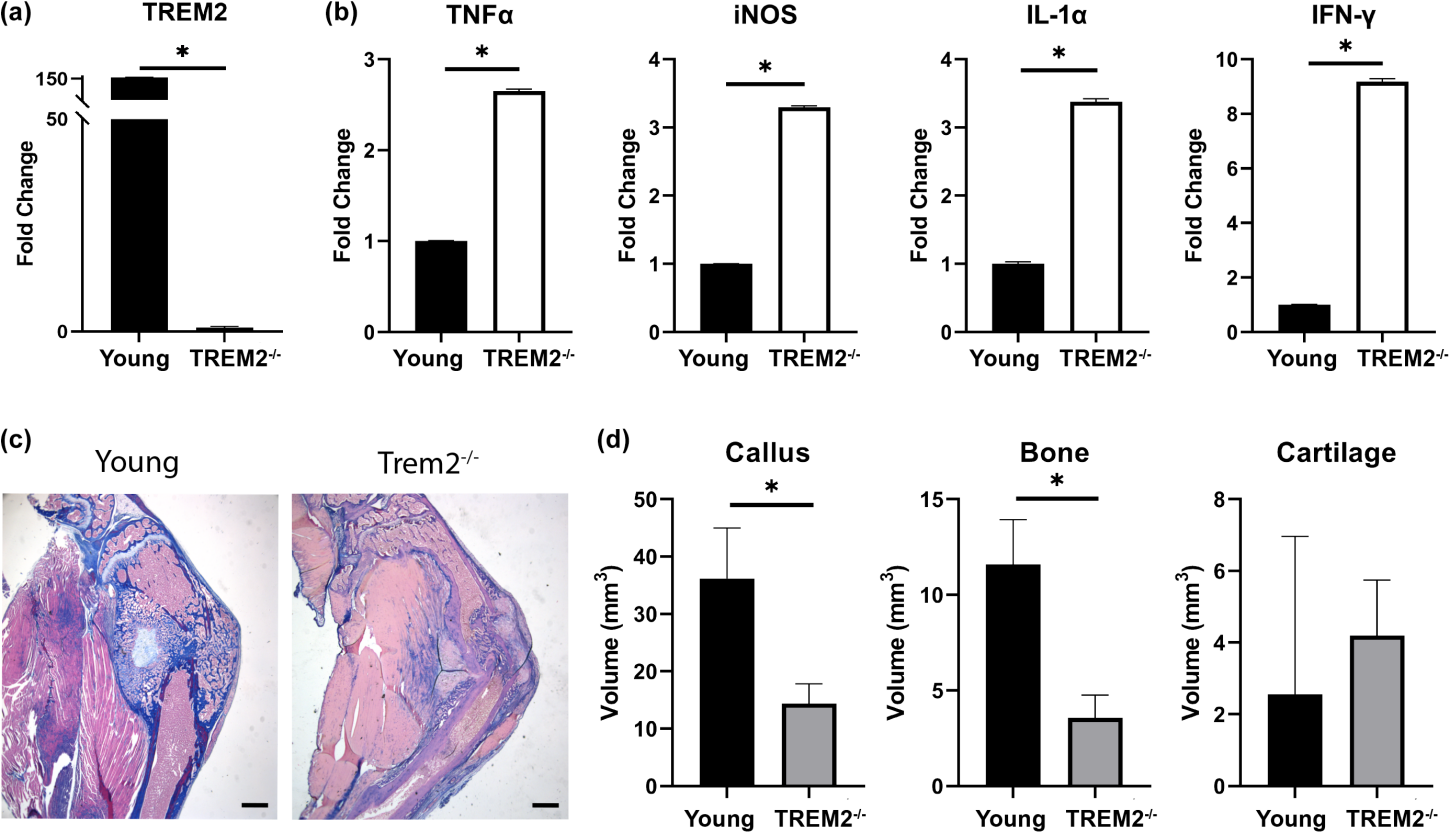
Inflammatory dysregulation and delayed fracture healing in Trem2‐null mice. (a) Bar graph comparing the fold change of Trem2 expression in the fracture callus of Trem2^−/−^ mice and aged‐matched young wildtype mice. Real‐time qPCR performed on callus tissue isolated 5 days post fracture (*n* = 5/group). (b) Bar graphs comparing the fold change of inflammatory cytokine expression in the fracture callus of Trem2−/− mice and aged‐matched young wildtype mice. Real‐time qPCR performed on callus tissue isolated 5 days post fracture (*n* = 5/group). (c) Representative histological sections of the healing fracture callus 10 days post fracture in young wild type and Trem2^−/−^ mice (scale bar = 1 mm). (d) Bar graph of the stereological analysis of the volume of callus and tissues at 10 days post fracture (*n* = 5/group). *(p < 0.05).

Finally, we wanted to better understand the role of Trem2 in fracture repair and the implications of decreased Trem2 expression in old mice. We obtained Trem2^−/−^ male mice (C57BL/6 background) originally generated and made available to us by M. Colonna (Turnbull et al., 2006). We have previously demonstrated the absence of Trem2 protein in the Trem2^−/−^ mice (Kawabori et al., 2015), and we further validated the absence of Trem2 transcripts in the fracture callus of Trem2^−/−^ mice via qPCR (Figure 6a). Tibia fractures were made in Trem2^−/−^ and aged‐matched control mice (4 months) as described here. At Day 5 post fracture, the calluses were isolated, and inflammatory cytokine expression was evaluated in the tissue via qPCR. The Trem2^−/−^ mice had significantly increased expression of pro‐inflammatory cytokine expression (TNFα, iNOS, IL‐1α, and IFN‐γ) compared to the aged‐matched control mice (Figure 6b). We then evaluated the extent of fracture healing in Trem2^−/−^ and aged‐matched control mice via stereology. Histological sections of the fracture callus were evaluated at 10 days post fracture, and the volume of the bone and cartilage within the callus was quantified (Figure 6c). The Trem2^−/−^ mice showed attenuated fracture healing with significantly decreased callus size and bone volume at Day 10 post fracture compared to the age‐matched controls (Figure. 6d). Interestingly, inflammatory dysregulation and attenuated fracture healing in the Trem2^−/−^ mice was similar to the fracture healing response in old mice (Figure 1a,b). Since the old mice had decreased Trem2 levels during fracture healing, these findings support the importance of Trem2 during fracture healing and detrimental effect of age‐related dysregulation of Trem2.

## 3 DISCUSSION

In this study, we profiled the cellular immune response during the early stages of fracture healing in old and young mice. We identified multiple transcriptionally distinct macrophage subpopulations present simultaneously within the fracture callus. Further analysis showed that macrophages from the callus of old mice had a more pro‐inflammatory phenotype compared to macrophages from young mice. The pro‐inflammatory macrophage phenotype in old mice was associated with local inflammatory dysregulation in the fracture callus tissue and perturbed fractured healing outcomes in old mice. Finally, we identified an age‐related decrease in Trem2 expression by macrophages from old mice. Knock out of Trem2 in young mice resulting in local inflammatory dysregulation and attenuated fracture repair, similar to phenotype in old mice. From these findings, we propose that Trem2 is an important regulator of inflammation during fracture healing and the age‐related alteration of Trem2 expression may be associated with the inflammatory dysregulation and perturbed fracture healing in old animals.

This work replicated our previous findings (Clark et al., 2020; Lu et al., 2005; Xing, Lu, Hu, Miclau, et al., 2010), and the work of others (Baht et al., 2015; Lopas et al., 2014), showing attenuated fracture healing in old mice compared to young, and further demonstrates the reproducibility of this animal model in the aging biology field. We further demonstrated that attenuated fracture healing was associated with local inflammatory dysregulation in the fracture callus. To gain a better understanding of the cellular immune response regulating inflammation during fracture healing, we utilized scRNAseq to analyze all Cd45+ immune cells isolated from the fracture callus of old and young mice. Similar to our previous work (Clark et al., 2020), we found that the number of immune cells infiltrating the fracture callus was similar in old and young mice. These findings suggest that the inflammatory dysregulation in old mice during fracture healing was not due to differential recruitment of immune cells and suggests that the age‐related differences may be more qualitative and better assessed via transcriptional analysis.

The scRNAseq analysis revealed age‐related transcriptional changes across all immune cell types analyzed within the fracture callus. Others have similarly demonstrated age‐related transcriptional changes of immune cells within multiple tissue types using scRNAseq including brain (Ximerakis et al., 2019), kidney (Almanzar et al., 2020), and lungs (Angelidis et al., 2019). This study did not provide detailed analysis of all immune cell types that were analyzed via scRNAseq. The focus of this paper was on the age‐related changes of macrophages during fracture healing, because we and others have previously implicated macrophages as critical for fracture healing (Chang et al., 2008; Clark et al., 2020; Schlundt et al., 2018). However, other immune cells, such as T cells B cells (Baht et al., 2018; Khassawna et al., 2017), have also been shown to have important roles in bone fracture healing, and it is possible age‐related changes in cells other than macrophages may also contribute to fracture healing outcomes. The raw data produced in this study may be a valuable resource for future analysis of age‐related changes to other immune cells affecting fracture healing outcomes.

In focusing this work largely on macrophages, we used scRNAseq to identify multiple macrophage subpopulations simultaneously present within the fracture callus. The analysis of macrophage subpopulations identified transcriptionally distinct subpopulations that suggested heterogenous activity and phenotypes across the subpopulations. The majority of the subpopulations were enriched for recruited macrophage markers and enriched for immune processes involved in the propagation of inflammation such as positive regulation of cytokine production, leukocyte chemotaxis and migration. Such findings are consistent with the pro‐inflammatory events of early fracture healing (Loi et al., 2016). However, there was variation among macrophage subpopulations, most notably on the extent of expression of pro‐inflammatory or recruited macrophage marker genes. Additionally, biological processes and pathways were differentially enriched among subpopulations, with some demonstrating immune surveillance processes while others were enriched for process involving propagation of inflammation. Phenotypic variation of macrophages as a function of health, disease, or tissue type has been the focus of a large body of research (Sreejit et al., 2020).

There is a growing understanding of the important role of tissue resident macrophages in health and disease. Tissue resident macrophages have been well characterized in many tissue types, including brain, liver, and skin, and have been described to have distinct functions and developmental origins that differentiate them from the infiltrating macrophages that are present in tissue after infection or injury (Sreejit et al., 2020). A tissue resident macrophage within bone has been previously reported (Chang et al., 2008); however, the field is still lacking adequate characterization of a bone resident macrophage. We found two macrophage subpopulations that did not express high levels of recruited macrophage markers and were not enriched for inflammatory propagating processes (clusters 5 and 6). These findings alone do not suggest that these two subpopulations were tissue resident macrophages within bone, as this study was not designed to identify these cells directly. Instead, these findings further demonstrate the heterogenous macrophage phenotypes present during fracture repair.

We applied multiple approaches to characterize the differences in macrophages within the fracture callus between young and old mice. The scRNAseq analysis demonstrated age‐related transcriptional changes were not equal among all subpopulations, with some subpopulations of macrophages appearing more resistant to age‐related transcriptional changes. The functional role of each subpopulation needs to be further validated to understand the specific effects of age on each subpopulation. However, we showed that an age‐related expansion of one macrophage subpopulation (cluster 3) was enriched for fibrotic‐associated macrophages. An enhancement of fibrosis can perturb proper regeneration of tissue, including bone.

The bulk RNAseq experiment was used in this study in order to expand the sample size and increase power analysis of fracture callus macrophages from old and young mice. We incorporated a previously generated data set from our group that had used bulk RNAseq to analyze the effect of inhibiting macrophage recruitment during fracture healing in old mice (Clark et al., 2020). Here, both scRNAseq and bulk RNAseq similarly revealed that age‐related changes shifted the transcriptional profile of macrophages to a more pro‐inflammatory phenotype in old mice compared to young. During fracture healing, the inflammatory process must be tightly regulated. Thus, more pro‐inflammatory macrophages in old animals may contribute to the inflammatory dysregulation and perturbed fracture healing. Such pro‐inflammatory phenotypes in old mice is consistent with a broad body of research that describes age‐related inflammatory dysregulation contributing to a myriad of pathologies that become more prevalent with increasing age (Xia et al., 2016). Pathological changes to macrophages have previously been implicated in other age‐related disease including Alzheimer's disease (Rawji et al., 2016), inadequate wound healing (Kim & Nair, 2019), and cardiovascular disease (Oishi & Manabe, 2016). The bulk RNAseq analysis was performed on F4/80+ macrophages isolated via a strict gating control via flow cytometry. The scRNAseq identified macrophages using marker genes that allowed for a broader characterization, including subpopulations of macrophages that may have diverged away from the more classic markers of macrophages. Therefore, it is possible that the bulk and scRNAseq analysis may not have analyzed identical populations of macrophages. However, the age‐related changes in macrophages were similar across both methodologies.

An interesting finding in our transcriptional analysis of old and young macrophages was the age‐related decrease in Trem2 expression. Trem2 has been shown to regulate inflammation in macrophages and has also been implicated in age‐related disease (Turnbull et al., 2006). A large body of literature has demonstrated a strong association of certain Trem2 alleles with Alzheimer's disease and related dementias (Carmona et al., 2018). Interestingly, a rare loss of function mutation of Trem2 in humans results in a condition known as Nasu‐Hakola disease (Dardiotis et al., 2017). This condition is characterized by early on set dementia and frequent bone cyst formation. While an understanding of Nasu‐Hakola disease has led to a focus on Trem2 in Alzheimer's disease and dementia, there has been minimal focus on the role of Trem2 within bone. The age‐related decrease in Trem2 expression within the fracture callus was validated via bulk RNAseq, qPCR, and flow cytometry. The scRNAseq analysis demonstrated a trend for decreased Trem2 expression in the old mice compared to young but differences were not statistically significant. The small sample size within each age group (*n* = 2) likely limited the statistical power for this comparison in the scRNAseq analysis. In mouse knockout models, we found that the absence of Trem2 resulted in local inflammatory dysregulation and perturbed fracture healing, similar to what was observed in old mice. To our knowledge, this is the first study to implicate the role of Trem2 on macrophages in fracture healing.

This work provides further support of the important role of macrophages in fracture healing and provides a detailed transcriptional characterization of the cellular immune response during fracture healing. A robust characterization of the age‐related differences in macrophages can also provide potential therapeutic targets in future studies that aim to alleviate the pathological burden of aging macrophages. Finally, this work has provided the first evidence of the important role Trem2 in fracture healing and demonstrated the need to focus future research on Trem2 in age‐related disease outside of Alzheimer's disease and related dementias.

## 4 METHODS

### 4.1 Animals

All procedures were approved by the UCSF Institutional Animal Care and Use Committee and conducted in accordance with the ARRIVE Guidelines for reporting animal experiments. Male mice (C57BL/6) were obtained from the National Institute on Aging Aged Rodent Colony. Mice were utilized for experiments at 24 months (old adult mice) or 4 months (young adult mice) of age. Trem2^−/−^ mice were originally generated by M. Colonna (Washington University, St. Louis, MO) and made available to us. Trem2^−/−^ mice were backcrossed for 5 generations. The genotype was authenticated by our group previously (Kawabori et al., 2015) and further validated here via qPCR (Figure 6a). Trem2^−/−^ mice were used at 4 months of age.

### 4.2 Tibia fractures

Young, old and Trem2^−/−^ were anesthetized and subjected to closed, non‐stable fractures of the tibia created by three‐point bending, as previously described by our group (Xing, Lu, Hu, Yu, et al., 2010). Analgesics were administered post‐surgery and no fixation device was utilized. The fractured tibiae were harvested on Day 3 post fracture for cell isolation from the callus, on Day 5 for qPCR analysis of the callus, or on Day 10 post fracture for stereological analysis.

### 4.3 Flow cytometry

Fractured tibiae were collected from old and young mice at Day 3 post fracture. The callus was dissected and the bone marrow was flushed with cold PBS to remove potential contaminating bone marrow cells from the callus‐infiltrating cells that were of interest. The callus tissue was first dissociated manually through a nylon cell strainer and further digested with Collagenase type I (0.2 mg/mL; Worthington, Lakewood, NJ) for 1 h at 37°. Cells were then isolated and resuspended in incubation buffer (0.5% BSA in PBS). Isolated cells were blocked and then stained with directly conjugated fluorescent antibodies: CD45 (clone 30‐F11), CD11b (clone M1/700), F4/80 (clone BM8), CD3 (145‐2C11), B220 (RA3682), NK1.1 (PK136), and Ly6G (clone 1A8), and TREM2 (clone 6E9) (Biolegend, San Diego, CA). Fixable Red Dead (Thermo Fisher, Waltham, MA) staining was used to gate for dead cells. Isotype controls and fluorescence minus one controls were used to gate for background staining. Cells were sorted on a FACSAria (BD Biosciences, San Jose, CA) at the San Francisco VA Healthcare System Flow Cytometry Core Facility, and were analyzed using FlowJoX software (Treestar). Cd45+ cells were sorted and used for single cell RNA sequencing. For bulk RNA sequencing, CD3+, B220+, NK1.1+, and Ly6G+ cells were excluded and CD45+, CD11b+, F4/80 were collected. FlowJo Software 9.6 (Treestar, Ashland, OR) was used for analysis.

### 4.4 Single cell RNA sequencing

Cells were isolated from the fracture callus of old (*n* = 2) and young (*n* = 2) mice at Day 3 post fracture and sorted via flow cytometry as described above. Viable CD45+ cells were isolated and prepared in single cell suspension. Library preparation was performed using the Chromium Single Cell 3′ Reagent Version 2 Kit by 10X Genomics. Cells from each sample were loaded into their respective 10X chip wells. Droplets were subjected to reverse transcription and then cDNA amplified. After quantification, libraries were prepared according to 10X Genomics guidelines with libraries sequenced on an Illumina HiSeq2500. Raw sequencing data has been uploaded to NCBI GEO (https://www.ncbi.nlm.nih.gov/geo/) with the accession number GSE198666.

### 4.5 Single cell RNA analysis

Sequencing data was merged from 10X Genomics Cell Ranger software into the R package Seurat (version 3.1.2) for further analysis. The SCTransform normalization method was used to control for between‐sample sequencing depth variation. Cells were removed from further analysis if they were identified to have <300 genes/cell or >3000 genes per cell, >20,000 UMIs/cell, >5% mitochondrial content, <0.1% hemoglobin content. The effects of mitochondrial content, ribosomal content, cell cycle, and sample/lane were regressed out prior to clustering. Genes were excluded from the final dataset if they were expressed in fewer than three cells. For differential expression analysis between clusters, genes were detected if they are expressed in at least 10% of cells in a cluster with a log fold change of at least 0.25. For DEG analysis between comparison groups, genes were detected if expressed in at least 5% of cells in the group with a log fold change of at least 0.25. Cell types were identified by the expression of marker genes that defined each immune cell type and visualized in violin plots and feature plots generated in Seurat.

### 4.6 Bulk RNA sequencing and analysis

Cells were isolated from the fracture callus of old (*n* = 10) and young (*n* = 11) mice at Day 3 post fracture and macrophages were sorted via flow cytometry as described above. RNA was extracted from the isolated macrophages using Invitrogen RNA aqueous Micro Kit (AM1931). TruSeq Stranded mRNA Library Prep Kit was used to prepare the library and Single‐end 50 bp sequencing was performed on Illumina HiSeq 4000. Reads were aligned to the mouse genome using STAR_2.4.2a (Ensemble Mouse GRCm38.78). DESeq2 was used to carry out the desired pairwise comparisons between groups.

### 4.7 Tibia processing and analysis of healing via stereology

The fractured tibiae were collected from old, young, and Trem2^−/−^ mice (*n* = 6/group) at Day 10 post fracture to analyze and compare the extent of healing via stereology. Isolated tibiae were fixed in 4% paraformaldehyde for 24 h and then decalcified with 19% ethylenediaminetetraacetic acid (pH 8) for 21 days with the solution changed every other day. After decalcification the tissue was prepared and embedded in paraffin. Serial sagittal sections (10 μm) of the fractured tibia and callus were mounted on slides and stained using Hall Brunt Quadruple Stain (HBQ) to visualize bone and cartilage. Total callus, bone and cartilage volume was quantified using an Olympus CAST system (Center Valley, PA) and software by Visiopharm (Hørsholm, Denmark) according to stereological methods developed by Howard and Reed (Cruz‐Orive, 1999) and described by our group previously (Xing, Lu, Hu, Miclau, et al., 2010).

### 4.8 Quantitative real‐time PCR

Young, old, and Trem2^−/−^ mice (n = 5/group) were sacrificed 5 days post fracture for quantitative real‐time PCR analysis. The whole callus was dissected and placed in Trizol and homogenized. RNA extraction was performed with isopropanol. cDNA was synthesized and quantitative real time PCR was performed on biological and technical triplicates. Relative gene expression of IL‐1α, TNFα, iNOS, IFNγ, and TREM2 was quantified. GAPDH was used as the house keeping gene.

Primers:

GAPDH (F:5′‐AGCCTCGTCCCGTAGACAAAAT‐3′, R:5′‐CCGTGAGTGGAGTCATACTGGA‐3′),

IL‐1α (F:5′‐AGTGCTGCTGAAGGAGATGCCTGA‐3′, R: 5′‐CCCCTGCCAAGCACACCCAGTA‐3′),

TNFα (F: 5′‐TGCCTATGTCTCAGCCTCTTC‐3′, R: 5′‐GAGGCCATTTGGGAACTTCT‐3′),

iNOS (F: 5′‐GAAGGGGACGAACTCAGTGG‐3′, R: 5′‐ GTGGCTCCCATGTTGCATTG‐3′),

IFNγ (F:5′‐CAGCTCCAAGAAAGGACGAAC‐3′, R:5′‐GGCAGTGTAACTCTTCTGCAT‐3′),

TREM2 (F:5′‐AAAGTACTGGTGGAGGTGCTG‐3′, R: 5′‐AGGCTAGAGGTGACCCACAG‐3′).

### 4.9 Statistics

GraphPad Prism v.7 software was used for analysis. Comparisons between groups was made by first using a two‐way ANOVA multiple comparisons test followed by a two‐tailed Student's *t*‐test. *p* < 0.05 was considered statistically significant. Differential gene expression was considered significant at FDR<0.1. For term enrichment in gene ontology and KEGG pathway analysis the level of significance was set using a modified Fisher Exact *p*‐value of *p* < 0.05.

## AUTHOR CONTRIBUTIONS

DC: conceptualization, methodology, formal analysis, investigation, writing—original draft, visualization. SB: methodology, validation, formal analysis, investigation. TM: resources, writing—review and editing, supervision, funding acquisition. SP: methodology, investigation, visualization. CLH: methodology, supervision, formal analysis, writing—review and editing. MN: conceptualization, formal analysis, resources, funding acquisition. RM: conceptualization, formal analysis, resources, writing—review and editing, project administration, funding acquisition.

## FUNDING INFORMATION

This work is supported by National Institutes of Health (NIDCR) grant number K08DE029505 (DC), the Department of Veterans Affairs Biomedical Laboratory R&D Merit Award BX002690 and NIH RO1NS132595 and RO1AA0027074 (CLH).

## CONFLICT OF INTEREST STATEMENT

The authors declare no conflict of interest.

## Supporting information


Table S1.

Table S2.

Table S3.

Table S4.

Data S1.

## Data Availability

Raw sequencing data has been uploaded to NCBI GEO (https://www.ncbi.nlm.nih.gov/geo/) with the accession number GSE198666.
