# Supplementary material for: Age‐related changes to macrophage subpopulations and TREM2 dysregulation characterize attenuated fracture healing in old mice
Source: Aging Cell. 2024 Jun 2;23(9):e14212. doi: 10.1111/acel.14212 (PMC11488338; doi:10.1111/acel.14212)
Supplement: Supplementary file 5 — Data S1. [file ACEL-23-e14212-s005.docx]

Table S1: Age-related DEGs within each cell type.

Table S2: Marker genes for each macrophage subpopulation

Table S3: Age-related DEGs within macrophage subpopulations

Table S4: Age-related DEGS within macrophages analyzed via Bulk RNAseq
